# Latissimus Dorsi Musculocutaneous Flap as a Therapeutic Option in Breast Reconstructive Surgery: A Case Report

**DOI:** 10.7759/cureus.69481

**Published:** 2024-09-15

**Authors:** José L Villarreal-Salgado, Alexis Ortega-Fernández, Carolina Montenegro Jimenez, Fernando Carballar Mejía, Sergio E Vázquez-Lara

**Affiliations:** 1 Plastic and Reconstructive Surgery, Instituto de Seguridad y Servicios Sociales de los Trabajadores del Estado (ISSSTE), Zapopan, MEX; 2 General Surgery, High Specialty Medical Unit 71, Torreon, MEX; 3 Surgical Gastroenterology, Hospital Regional Instituto de Seguridad y Servicios Sociales de los Trabajadores del Estado (ISSSTE), Guadalajara, MEX; 4 General Surgery, Instituto de Seguridad y Servicios Sociales de los Trabajadores del Estado (ISSSTE), Monterrey, MEX

**Keywords:** autologous breast reconstruction, chestwall reconstruction, latissimus dorsi flap, plastic surgergy, sarcoma soft tissue

## Abstract

In plastic surgery, the reconstructive ladder is a systematic approach used to guide the planning and execution of reconstructive procedures. The use of autologous tissues is preferred in the breast reconstruction process due to the multiple advantages they offer. The latissimus dorsi musculocutaneous flap has been the therapeutic option of choice in these cases, replacing other surgical techniques because of the high adaptability and low complication rates. We report a case of a 69-year-old female who presented a lesion in the right breast reported as sarcoma, who underwent into an extended resection and reconstructive procedure with a latissimus dorsi musculocutaneous flap.

## Introduction

The latissimus dorsi muscle is a flat and triangular muscle located in the lower back. Functionally, this muscle is responsible for arm and shoulder movements, including extension, adduction, and medial rotation of the humerus. In breast reconstruction, the use of autologous tissues is preferred, especially after radiotherapy and when local tissues are of poor quality [1]. A proper surgical technique seeks to increase soft tissue coverage with the flap, allowing future interventions and minimizing complications in the donor area [2]. The latissimus dorsi musculocutaneous flap is widely used because of its ability to provide enough tissue, achieving good volume and contralateral symmetry, maximizing the aesthetic result [3]. Due to the severity and infrequency, in this article, we present the case of a breast reconstruction using the latissimus dorsi flap with the aim of presenting our management and updating the information in the literature.

## Case presentation

**Figure 1:**
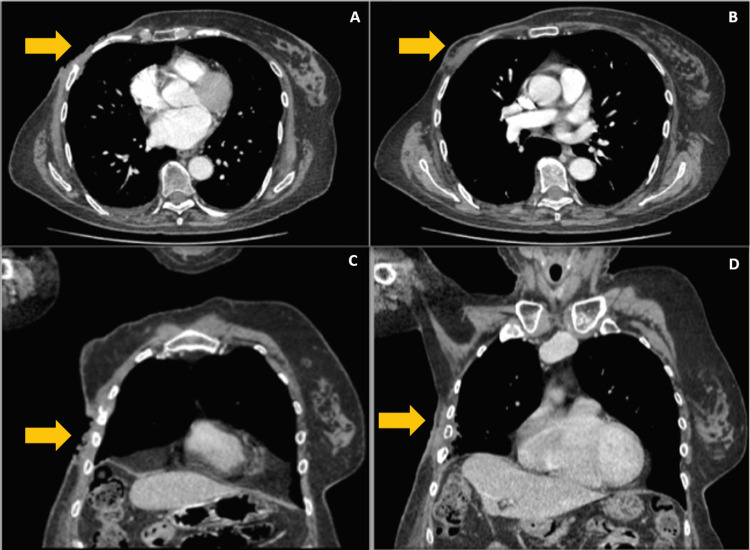
Contrasted CT scan of the thorax shows the mass on the right breast and costal archs (A-B, axial view; C-D, coronal view) The yellow arrow points the area of depth of the mass.

A 69-year-old female with a history of systemic arterial hypertension, smoking, and alcoholism was diagnosed in 2002 with breast cancer treated with chemotherapy, radiotherapy, and radical mastectomy of the right breast. Afterwards, in November 2023 she noticed a single and painless lesion in the right breast region. One year later the patient went for medical evaluation. Physical examination revealed tenderness in the right breast accompanied by an increase in the size of the lesion, the presence of serous exudate, and unintentional weight loss. As the treatment needed to be initiated, it was managed with empiric antibiotics without improvement. An excisional biopsy was performed reporting a mesenchymal tumor (sarcoma). A simple and contrasted CT scan of the thorax (Figure 1A-1D) showed a solid mass of 50 x 52 x 10.8 mm in soft tissues covering the topography of the right mammary gland producing ulceration of the skin. Also, the 4th, 5th, and 6th anterior costal arches were involved in producing destruction of the cortical layer; changes at the pulmonary parenchyma level with fibrotic bands were also reported.

**Figure 2:**
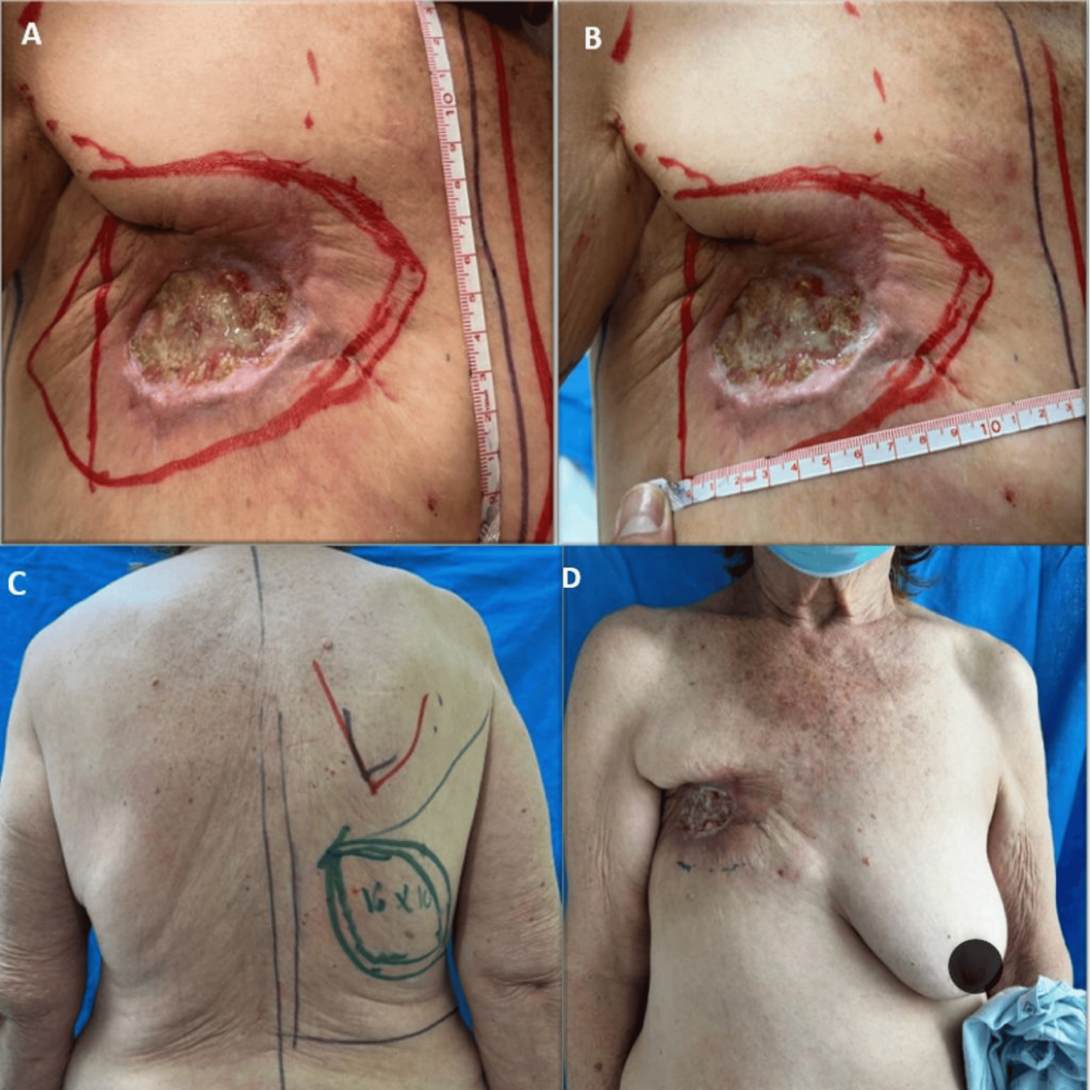
Marking of the lesion prior to surgical procedure (A-C, design of latissimus dorsi musculocutaneous flap; D, ulcerated lesion in the right anterior chest wall)

Three doses of denosumab 120 mg/month were applied prior to surgery. Pre-surgical assessment was performed to plan the optimal reconstructive procedure. Physical examination of the thoracic region showed a 6 x 4 cm ulcerative lesion in the anterior and lateral right mammary region with erythematous borders and the presence of serous exudate. Marking was made prior to the surgical procedure (Figure 2A-2C).

**Figure 3:**
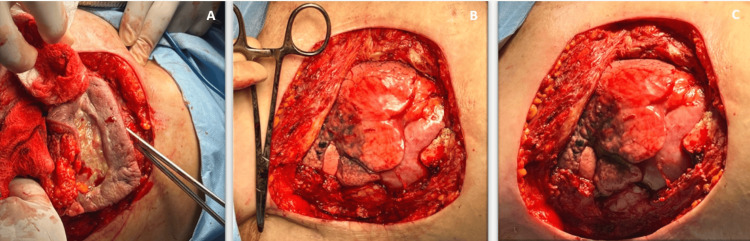
Surgical approach of the mass (A-C, defect in the anterior wall of the right hemithorax)

An approximate measurement of 11 x 9 cm was determined in the defect of the anterior wall of the thorax, so based on this measurement the design of the latissimus dorsi flap was made. Oncological surgery performed the resection of the lesion (Figure 3A-3C), covering the entire thickness of the right hemithorax, including part of the anterior costal arches.

**Figure 4:**
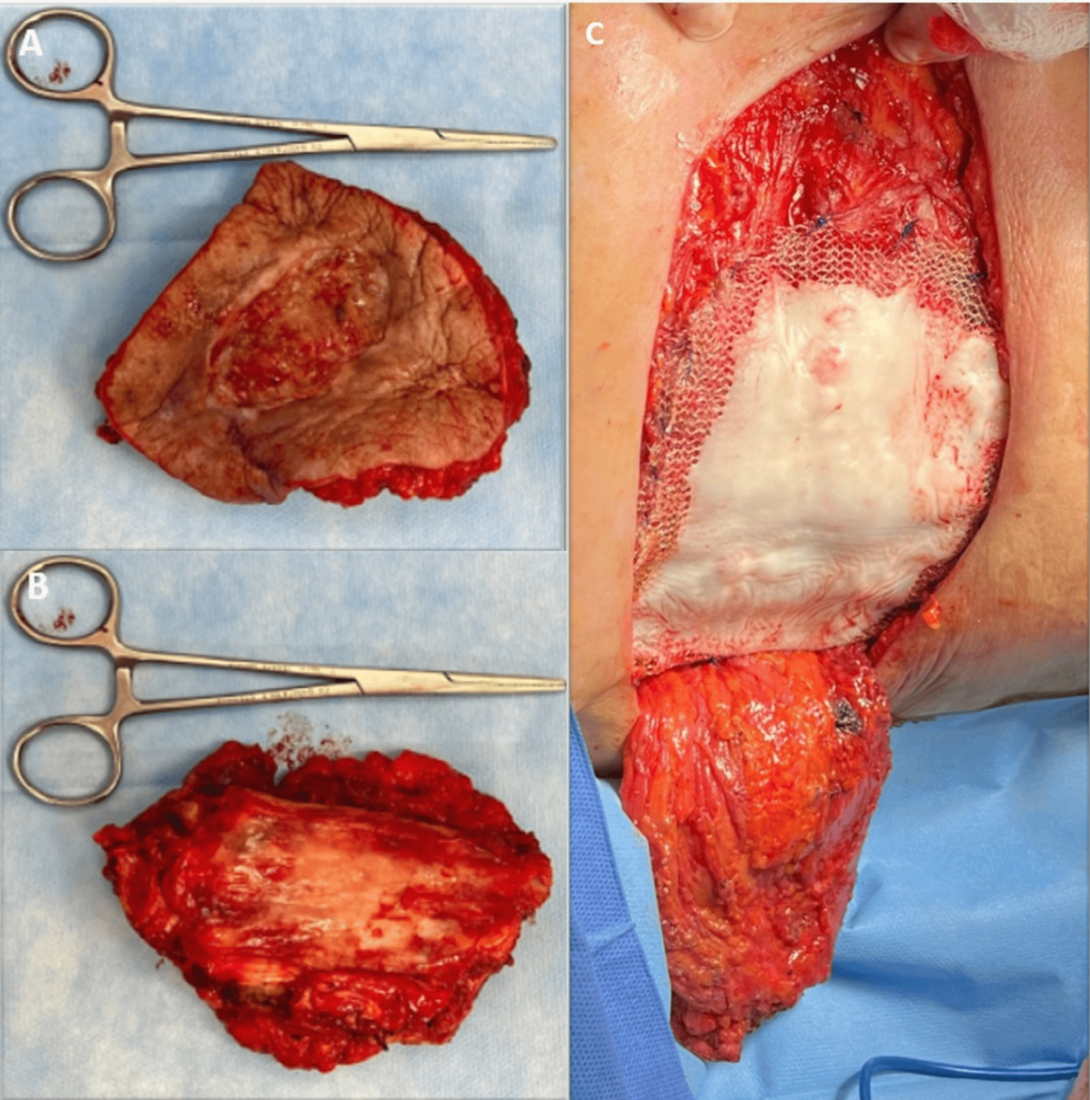
A-B, resected specimen of the right hemithorax including part of the anterior costal arch; C, defect coverage with polypropylene mesh

A polypropylene mesh with tobramycin was placed to close the wall defect. Fixation was made with non-absorbable suture (Figure 4A-4C).

**Figure 5:**
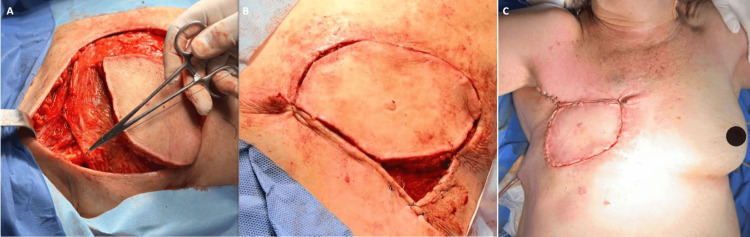
Latissimus dorsi flap technique (A, latissimus dorsi muscle attached to pectoralis major muscle; B, cutaneous part of the flap attached to the skin of the defect area; C, reconstruction of the anterior thoracic wall)

A latissimus dorsi flap was performed as a technique for chest wall reconstruction (Figure 5A-5C).

**Figure 6:**
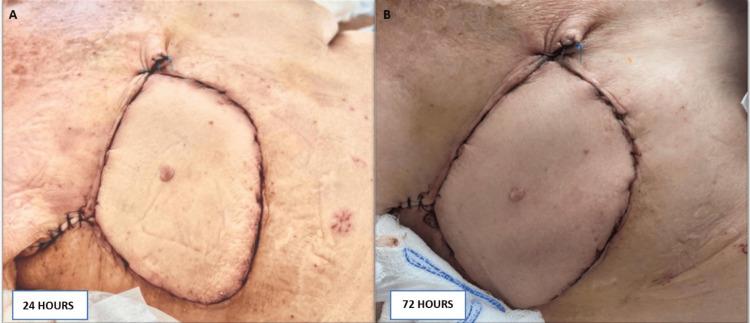
Evaluation of the latissimus dorsi flap during postoperative period (A, flap result 24 hours after surgical procedure; B, flap result 72 hours after surgical procedure)

With the patient placed in lateral decubitus, the anterior wall of the thorax was dissected. In the dorsal area, the skin island was incised until the muscle surface was identified and removed from the muscular plane in the proximal and distal directions. Approximately 8 cm of dorsal muscle was exposed and included in the flap. The upper border of the latissimus dorsi was lifted identifying the thoracodorsal fascia. The costal insertions were dissected downwards. The intercostal perforators were ligated and sectioned. The dissection is advanced forward being careful not to injure the serratus major, which was deep to the latissimus dorsi. The vascular branches of the serratus major muscle and the scapular circumflex artery were ligated and sectioned giving more length to the pedicle. The dissection of the latissimus dorsi was completed, remaining only attached to the axillary region. The dorsal region wound was closed by planes. Subsequently, the patient was placed in a supine position. The latissimus dorsi muscle is extended without any tension in the defect area. Fixation stitches were applied with a nonabsorbable suture that joined the latissimus dorsi muscle to the pectoralis muscle. The skin island of the latissimus dorsi flap was sutured to close the defect at its upper edge and fixed to the rest of its edges. During the postoperative period, the patient had a satisfactory evolution denying any symptomatology. The evaluation of the latissimus dorsi flap showed adequate perfusion at 24, 72, and 144 hours (Figure 6A-6B).

## Discussion

The greatest challenge in the treatment of thoracic sarcoma is the surgical removal of the tumor since it implies the resection of a significant part of the thoracic wall. For this reason, adequate communication between the oncologic surgeon and the reconstructive surgeon is of vital importance. It is essential for these patients to establish an appropriate preoperative plan, taking into account the extent of the resection and considering the reconstructive alternatives to avoid damaging potential muscle flaps. Chest wall reconstructive strategies seek to maintain the integrity of the intrathoracic organs and provide adequate stability to the chest wall while preserving its form and function. The thoracic skeleton can be stabilized using polypropylene or polytetrafluoroethylene (PTFE) meshes and titanium rods, although the use of dermal matrices and the combination of both has also been described [4-6].

The reconstruction of the chest wall defect has led to the use of various flaps such as latissimus dorsi, transverse rectus abdominal muscle (TRAM), pectoralis muscle, and others [7]. As for the latissimus dorsi muscle, its usefulness has been seen in various reconstructions, the skin island provides a strong dermis, which allows sutures between it and the surgical margins, in order to provide a tight closure of the chest cavity. The pectoralis muscle flap is a good alternative for reconstructing central defects of the thorax. It is usually used in large and central tumors at the sternal level [8]. The TRAM flap has demonstrated its versatility especially in thoracic defects located at the level of the breast, due to mastectomies related to tumors, offering the patient an alternative. However, although this flap is ideal for reconstructing central and lateral defects, it has a smaller arc of rotation than that provided by the latissimus dorsi flap. The latissimus dorsi flap has become the technique of choice because of its innumerable advantages over other reconstruction techniques, including immediate reconstruction of large defects, pedicled and free use, provision of large skin surfaces (up to 30 × 20 cm), large tissue volume, hairless skin, donor site that does not affect the patient's appearance, large arc of rotation, excellent mobility and 180-degree rotation [9].

## Conclusions

With this case, we can demonstrate that the latissimus dorsi flap is the best alternative for therapeutic management in cases of breast reconstruction since it is a safe method because it offers a reliable and consistent pedicle providing adequate vascularization to the recipient tissue. Likewise, this flap offers several advantages such as avoiding microvascular anastomoses, a reproducible surgical technique, versatility in the shapes and orientations that can be performed, among others. Undoubtedly, the low rate of morbidity and mortality associated with this procedure significantly contributes to improving patients' quality of life by reducing the physical and psychological impact of the process.
